# March

**Published:** 1890-03-01

**Authors:** 


### MARCH.

March, it is said, often comes in like a lion and goes out like a lamb, but the same observation has been made respecting at least one other month. A recent author observes that "the most enthusiastic lover of dear dirty London would wreck his reputation for veracity were he to declare that city a pleasant dwelling-place in March, with its biting winds and bitter rains." But dreary as the town appears when swept by east winds, especially on a Sunday, the country at this season of the year is quite as dreary when the east winds blow. Still, notwithstanding easterly biting winds, we are en route to a more congenial season. For on the 20th and 21st of March the sun is between Aries and Pisces. Exactly one-half of the earth is illuminated, and therefore the days and nights are equal. Nature now commences to rejoice in the final and complete victory of spring over winter. Yet March is a dangerous month, especially to invalids—more particularly so to weak-chested invalids. And it is not a time when those who have wintered abroad should return to this country. For although the mean temperature may be elevated, in round numbers, from 40 deg. to 55 deg., the warmth is diminished by the easterly and northerly winds which are so prevalent. Moreover, the temperature is very variable, increasing often by sudden rises from the succession of sunshine to previous cold, cloudy weather, and diminishing when the latter, as is so frequently the case, again usurps authority. Then the temperature suddenly falls, probably with showers, hail, sleet, and wind, and, in some instances, frost at night. Last March we heard a man remark, "It has done everything to-day except rain hot water!"

An old writer observed, "The young and joyous spirit of approaching spring sheds its sweet influence on everything, the streams sparkle and ripple in the noon-day sun, and the birds carol tipsingly their merriest ditties." And another ancient minstrel sang of a spring day that it

"Looks beautiful, as when an infant wakes
From its soft slumbers."

Nevertheless, we think the above in the present days is more applicable to May than to March. But then we are told that the seasons have changed, which, however, as pointed out in our article on February, is not supported by facts.

March 1st is the old St. David's Day, supposed to be so called because, in the time of King Arthur, Archbishop St. David won a great victory over the Saxons, having previously ordered his soldiers to place a leek, "the fairest emblym that is worne," in their caps by way of distinction. There are, however, other reasons given why "honest Taff should wear his leek." March 2nd is St. Chad's day, and St. Chad's Well, once at the end of Gray's Inn Lane, was famed for the curative properties of its waters. But an old author said, "The ingenious chemists can make as effectual water as Chad's Well at the small price of one halfpenny!" We should like to know how much of the so-styled natural mineral waters now consumed are made by chemists. March 12th is St. Gregory's Day, of whom many miracles are related, but not credited in this unbelieving age. In the "Yea and Nay Almanack" for 1673 the following receipt for sore eyes was given, to be used on the 12th of March: "As you rise, instead of fasting spittle, or a cat's tail, rub your eyes with a hundred broad pieces of your own gold, and I tell thee, friend it will not only do thy eyes good, but thy purse also." A prescription as likely to be adopted by the majority, as the advice of the physician of the present day, who tells an invalid destitute of means to winter on the Riviera, or to drink best port daily. March 16th was once noted as St. Finian's Day. St. Finian was surnamed "Lobhat," or the leper, and it appears he was credited with the power of turning wine into water, and the reverse. Unfortunately he was not more able to cure himself, than physicians are now capable of curing leprosy. On or about March 20th the cuckoo, "beauteous stranger of the grove, companion of the spring" is generally first heard—the mysterious cuckoo appearing from no one knows where, and, disappearing no one knows whither. A considerable amount of interest has been attached to this bird in consequence of its supposed habits, as we were taught in our childhood. According to Jenner, the cuckoo lays its eggs in other birds' nests, and when the young cuckoo comes forth it pushes the young or eggs of the real mistress of the nest over the side. And Jenner said it was anatomically framed to accomplish this feat. But recently all this interesting story has been exploded by Dr. Creighton.

In conclusion, we offer a little advice, which we trust may prove the exception to the rule of gratis advice not being followed. In a bright March morning, which we may have, do not be deluded into the idea that summer has come. Do not go abroad thinly clad. For on turning the street corner you may be met by a sharp east wind, which may seize your throat or chest or limbs, and then—why, perhaps you will be said to have the influenza! But it is a time of hope and expectation! There is a stir and sweetness of awakened life in the air; already certain flowers gem the black earth, trees begin to change their outline, as brown buds swell and burst. A little more patience, and you may cast off winter garments.

